# Antioxidant activity and cytotoxic effects of *Benincasa hispida* juice on HepG2 cells

**DOI:** 10.1186/s12906-026-05475-3

**Published:** 2026-07-25

**Authors:** Shi-Bo Wang, Ling-Xue Kong, Ji-Luan Zhang, Jin-Juan Liu

**Affiliations:** Key Laboratory of Biotechnology for Medicinal Plants of Jiangsu Province, School of Life Sciences, Jiangsu Normal University, Xuzhou, 221116 China

**Keywords:** *Benincasa hispida*, Antioxidant, Apoptosis, HepG2 cells, Caspase, Migration and invasion

## Abstract

*Benincasa hispida* Cogniaux is widely utilized as a dietary staple and a medicinal remedy in China owing to its diverse biological properties. However, its potential antioxidant and anticancer properties has rarely been systematically reported. Therefore, this study aimed to investigated the in vitro antioxidant and anticancer effects of *Benincasa hispida* juice (BHJ). The total phenolic and flavonoid contents of BHJ were determined to be 37.14 ± 4.26 mg GAE/L and 12.43 ± 2.36 mg RUT/L juice, respectively. BHJ at the original concentration (1×) exhibited potent scavenging activity against DPPH radical, superoxide anions(O_2_^−^), hydroxyl radicals(·OH) and nitrite, with respective scavenging rates of 43.15 ± 2.18%, 24.35 ± 2.37%, 19.57 ± 1.49% and 38.64 ± 3.42%. Treatment of HepG2 cells with BHJ at concentration of 10, 20, and 40 mL/L significantly induced cellular apoptosis, which was accompanied by upregulation of activated caspase 9, PARP, Bax, Bad, and Apaf-1, as well as downregulation of Bcl-2 and pro-caspase 3. Furthermore, caspase-specific inhibitors (Ac-DEVD-CHO and Z-LEHD-FMK) effectively abrogated BHJ- induced reduction in HepG2 cells viability. Additionally, BHJ at 5 and 10 mL/L significantly suppressed the migration and invasion of HepG2 cells. This anti-metastatic effect was associated with the downregulation of matrix metalloproteinase(MMP)-2 and MMP-9, along with the upregulation of TIMP-1. Collectively, these findings demonstrate the potent antioxidant and anticancer capacities of BHJ, suggesting that *Benincasa hispida* may serve as a promising source of therapeutic agents for combating oxidative stress and hepatocellular carcinoma.

**Supplementary Information:**

The online version contains supplementary material available at https://doi.org/10.1186/s12906-026-05475-3.

## Introduction

Hepatocellular carcinoma (HCC), one of the most aggressive malignancies, ranks fourth in incidence and second in mortality among all cancers in China [1]. Despite the availability of diverse treatment modalities(e.g., surgical resection, chemptherapy, and targeted therapy), the high post-surgical recurrence rate of HCC contributes to its status as the third leading cause of cancer-related deaths globally [2]. Therefore, the development of effective preventive and therapeutic agents specifically targeting HCC is of great significance.

Natural plant-derived products have garnered considerable attention as promising candidates for cancer prevention and therapy [3, 4]. Accumulating evidence has demonstrated that diets rich in fruits and vegetables are correlated with a reduced cancer mortality rate [5, 6]. Moreover, numerous phytochemicals isolated from these plants exhibit prominent antioxidant and anticancer properties [7]. Notably, combinations of phytochemicals often exert greater efficacy than individual components alone via additive or synergistic effects in cancer prevention [8]. This has promoted the investigation of whole phytochemical mixtures from fruits and vegetables for potential anticancer and antioxidant properties, as opposed to focusing solely on single molecules or fractions.

*Benincasa hispida* Cogniaux, a member of the Cucurbitaceae family commonly known as wax gourd or winter melon, is widely consumed as a vegetable in East Asian countries and has long been valued as a dietary therapeutic food in traditional Chinese medicine(TCM) due to its mild nature and nutritional richness [9]. As a typical medicine-food homology ingredient, wax gourd juice is frequently incorporated into daily diets for its purported health-promoting effects, such as clearing heat, promoting diuresis, and nourishing the body-properties that align with TCM’s emphasis on preventing disease through dietary regulation. Wax gourd is enriched in various bioactive phytochemicals, including phenolic acids, sterols, terpenes and flavonoids [10]. However, existing research on wax gourd’s bioactivity has several notable limitations: most studies have focused on its peel, seeds, or isolated components (e.g., triterpenoids, polysaccharides, or lectins) [11], while the biological effects of its juice—the most commonly consumed and easily accessible form in daily diets—remain largely uncharacterized.

Traditionally, wax gourd has been used in Chinese medicine for its anti-aging, analgesic, antioxidant, and anti-inflammatory properties [12, 13]. Previous research has reported that *Benincasa hispida* seed extract inhibits the proliferation human triple-negative breast cancer cell by inducing S-phase cell cycle arrest [14]. Additionally, Two chito-specific lectins from *Benincasa hispida* fruit extract trigger caspase-9 mediated apoptosis and early cell cycle arrest in human pancreatic cancer cells [15]. Polysaccharides derived from *Benincasa hispida* exhibited fruit have also been shown to exhibit antioxidant activity both in vitro and in vivo [16]. Furthernore, the aqueous peel extract of *Benincasa hispida* combined with selenium nanoparticles displayes potent anticancer activity against HeLa cells [17]. However, as a dietary staple and dietary therapeutic ingredient, the antioxidant and anticancer effects of *Benincasa hispida* juice remain poorly understood to date. Therefore, the present study aims to elucidate the potential of wax gourd juice as a therapeutic agent against HCC by evaluating its antioxidant activity ant its effects on HepG2 cells, thereby providing scientific evidence for its traditional dietary therapeutic value and supporting its development as a natural, safe, diet-derived preventive or adjuvant therapeutic option for HCC.

## Materials and methods

### BHJ preparation

The Chinese variant of wax gourd was procured from a local market, cleaned, deseeded and peeled. As per a previous method with minor modifications, BHJ was then extracted [18]. Briefly, BHJ was extracted using a household juicer and centrifuged at 1000 g at 4 °C for 30 min. The supernatant BHJ (original juice) was used as stock solution and stored in aliquots at -80 °C until further analysis. A series of working concentrations were prepared by serial dilution with cell culture medium for cytotoxicity assays.

### Reagents and chemicals

Cleaved-Caspase 9, pro-caspase 3 antibodies were from Cell Signal Technology (CST, CA). Bax, Bcl-2, Apaf-1, Bad, MMP-2, MMP-9, TIMP-1, GAPDH were from Bioworld Technology (Bioworld, USA). Z-LEHD-FMK was from BioVision (BioVision, CA, USA). PARP and Ac-DEVD-CHO was from Beyotime Institute of Biotechnology (Haimen, China). Unless stated otherwise, all other reagents were purchased from Sigma-Aldrich (St. Louis, MO).

### Phytochemical compounds contents

#### Total phenolic content

The total phenolic and flavonoid content was measured by the Folin-Ciocalteu method. Briefly, the total phenolic content in BHJ was calculated by comparison with a standard curve prepared with 0–200 µg/mL gallic acid. Total phenolic content was determined using an equation: y = 0.0028x + 0.0035 (y: absorption value; x: the concentration of gallic acid, R^2^ = 0.9989). The content of total phenolic in the extraction was expressed as gallic acid equivalent (GAE) per liter of BHJ.

#### Total flavonoid content

Total flavonoid content was measured using AlCl_3_ colorimetric assay. Briefly, the content of total flavonoid in BHJ was calculated using a standard curve prepared with 0–64 µg/mL rutin (y = 0.0116x + 0.0151, y: absorption value; x: the concentration of rutin, R^2^= 0.9975). The content of total phenolic and in the extraction was expressed as rutin equivalents per liter of BHJ.

### Antioxidant capacities

#### DPPH radical scavenging

The DPPH radical scavenging activity was determined as described previously [19]. Briefly, BHJ was mixed with 100 µL 0.1 mM DPPH, and incubated for 30 min. The absorbance was detected at 517 nm using a microplate reader (Bioteck, USA). The DPPH radical scavenging rate of the BHJ was indicated as the percent decrease in absorbance compared with control.

#### Superoxide anions (O_2_^−^ ) radical scavenging

The scavenging activity for O_2_^−^ radical was measured using the pyrogallol autoxidation method [19]. Briefly, 6mL Tris-HCl buffer (pH8.2) and 1 mL of BHJ was incubated in a reaction tube. After keeping it at 25 °C for 10 min, 1mL 8 mM pyrogallol solution was added, then mixed it quickly. Two drops of concentrated HCl solution (12 M) were add after 4 min of accurate reaction. The absorbance was measured at 325 nm and denoted as A_1_. The absorption of pure water in the reaction instead of the sample was denoted as A_0_. The O_2_^−^ radical clearance was calculated as follows equation: O_2_^−^ radical clearance rate (%) = (A_0_-A_1_)/A_0_ × 100%.

#### Nitrite scavenging

The nitrite scavenging activity was detected by the method of hydrochloric acid naphthalene ethylenediamine coloration [19]. 1 mL sample or 1 mL of 50% ethanol (blank) was mixed with 1 mL of 5 mg/L nitrite solution and 1 mL of citric acid buffer (pH 3). After reacting for 30 min at 37 °C, 1 mL of 4 g/L amino benzene sulfonic acid sodium (in 20% hydrochloric) was added in the mixture, and then 0.5 mL of 2 g/L hydrochloric acid naphthalene ethylenediamine (in water) was also added after 3 min. The mixture was reacted for 15 min and measured at 538 nm. Ascorbic acid was used as positive control. Nitrite-scavenging ratio (%)=(1 − A_sample_/A_blank_)×100%.

#### Hydroxyl radical scavenging

Hydroxyl (OH) radical scavenging activity of BHJ was detected as described previously [19]. Briefly, 1 mL BHJ was mixed with 2 mL 50 µM phosphate buffer solution (PBS, pH 7.4), 1.5 mL 5 mM 1,10-phenanthroline, 1 mL 7.5 mM FeSO_4_ and 1 mL H_2_O_2_ (0.1%) were added into a tube, the volume was 10 mL after the tube had been added ultra-pure water finally. Then the tubes were kept for 60 min at 37 ℃, and the absorbance at 536 nm was measured. Scavenging activity (%)=(A_2_-A_1_)/(A_0_-A_1_)×100%, where A_0_ is the absorbance value of the solution without the addition of samples and H_2_O_2_, A_1_ is the absorbance value of blank control without BHJ, and A_2_ is the absorbance value of the solution with BHJ.

### Proliferative inhibition

#### Cells proliferation assay

HepG2 cell line were from Shanghai Institute of Cell Biology, Chinese Academy of Sciences (Shanghai, China), and maintained in DMEM supplemented with 10% fetal bovine serum (FBS, Gibco, USA), 100 U/mL penicillin and 100 µg/mL streptomycin at 37 ℃ in a 5% CO_2_ incubator. The cell viability was determined using Alamar blue method [20]. Briefly, cells were inoculated into 96-well plates(Corning, USA) and treated with BHJ for 48 h. Thereafter, 200 µL alamar blue solution was added after washing with PBS and cultured for an additional 3–4 h. The absorbance was measured using microplate reader (Bioteck, USA) at λex = 530 nm and λem = 590 nm. The cell growth inhibition rate was calculated.

#### Colony formation

HepG2 cells were seeded in 6-well plates(Corning, USA). Then cells were treated with BHJ for 48 h, detached by trypsin and reseeded for colony formation for 2 weeks. The cells were stained with crystal violet for observation under microscope (Zeiss, Germany).

#### Hoechst33258 staining

HepG2 cells were seeded on the slides in 6-well plate(Corning, USA) with 500 cells per well and incubated with BHJ for 48 h, and were then fixed in 4% paraformaldehyde for 2 h. After washing with PBS, the cells then incubated with 50 µg/mL of Hoechst 33,258 staining for 30 min. Cells were observed using fluorescence microscopy(Corning, USA).

#### Flow cytometer analysis

HepG2 cells were seeded in 6-well plate(Corning, USA) with 3 × 10^5^ cells and treated with BHJ (10, 20, 40 mL/L) for 48 h, PBS as control. Then both attached and detached cells were harvested and treated according to the manufacturer’s directions (Beyotime Inc). Cells were washed twice with PBS, resuspended in binding buffer, stained first with FITC Annexin V, and then stained with PI before flow cytometer. Flow cytometer analysis was performed on a FACS Calibur (Becton Dickinson, San Jose, CA). For each analysis, a minimum of 10 000 cells was counted. The apoptosis rate of cells refers to the total number of cells in the second and third quadrants.

#### Caspase activity

The activities of caspase 9, caspase 3 were detected by the kit (Beyotime, China) follow the instructions. Briefly, after treatment with BHJ for 48 h, HepG2 cells were collected and lysed by lysis buffer. Lysates were incubated with pro-caspase 3 and Cleaved-caspase 9 substrate for 2 h. Samples were measured with a microplate reader (Bioteck, USA) at an absorbance of 405 nm. To test the activity of caspase 3 and caspase 9, 10 µM of Z-LEHD-FMK (BioVision, CA, USA) or 20 µM Ac-DEVD-CHO (Beyotime, Haimen, China) were added and incubated for 2 h prior to addition of BHJ for 48 h, then cell viability was detected by alamar blue assay.

#### Western blot analysis

Total proteins(control or BHJ treated for 48 h) were extracted and the expression of related protein was detected as previously described [20]. Equal amounts of protein were then immobilized using 5–12% SDS-PAGE and transferred onto PVDF membranes from Merck Millipore (Billerica, MA, USA). The membranes were blocked for 1 h and subsequently incubated overnight at 4 °C with antibodies against Bax, Bcl-2, Apaf-1, Bad, MMP-2, MMP-9, TIMP-1, PARP, and GAPDH. Afterward, the membrane was incubated with horseradish peroxidase (HRP)-conjugated secondary antibodies for 2 h. Following three washes with TBST, detection was carried out using a chemiluminescence detection system from Protein Simple (USA). The intensity of the western blot bands was quantified, and the values were expressed relative to that of GAPDH.

### Inhibiting migration and invasion capacities

#### In vitro wound-healing assay

HepG2 cells were plated at a density of 6 × 10^3^ cells/well in a 6-well plate(Corning, USA) and grown to confluence. Next, by using a sterile plastic yellow tip, a scratch was created in the monolayer. To remove damaged or floating cells, the medium was then removed, and the cells were washed with PBS. The cells were treated with 5 and 10 mL/L BHJ for 24–48 h. The plates were then examined by light microscopy(Corning, USA) for their migration ability. All assays were performed in triplicates.

#### Transwell assay

The cells were determined using a transwell assay with or without Matrigel (Corning, NY, USA). In brief, the cells were starved overnight. The cells at a density of 2 × 10^5^ cells/mL were seeded with Matrigel into the upper chamber of the transwell. The upper chamber was filled with RPMI-1640 medium without FBS. RPMI-1640 medium containing 15% FBS was plated into the lower chamber of the transwell. The transwell was maintained at 37℃ for 36 h (without Matrigel) or 48 h (with Matrigel), allowing the cells to invade into the lower chamber. The invaded cells were harvested and then fixed with 4% paraformaldehyde at 4℃ for 30 min. The cells were then stained with 0.1% crystal violet dye for 20 min at room temperature. The cells were observed under an microscope(Zeiss, Germany).

### Statistical analysis

Statistical analyses were performed with GraphPad Prism version 8.0 (GraphPad Software, Inc). Experimental data were analyzed using one-way ANOVA. The significance level was set as *P* < 0.05, and was noted when *P* < 0.01. Data was expressed as mean±standard error, and derived from three independent experiments.

## Results

### Phytochemical contents in BHJ

Phytochemicals such as phenolics and flavonoids are well-recognized for their potential antioxidant and anticancer activities. Therefore, we first quantified the total phenolic and flavonoid comtents in BHJ using established colorimetric methods. As summarized in Table 1, the total phenol(TPC) of BHJ was determined to be 37.14 ± 4.26 mg GAE/L juice, while the total flavonoid content(TFC) was 12.43 ± 2.36 mg RUT/L juice.

**Table 1:** The contents of total phenol and flavonoid in BHJ

Sample	Total phenolic contents (mg GAE/L)	Total flavonoid contents (mg RUT/L)
BHJ	37.14 ± 4.26	12.43 ± 2.36

### Radical scavenging analysis of BHJ

To evaluate the antioxidant potential of BHJ, we systematically determined its scavenging activity against these radicals and nitrite using established in vitro assays. As shown in Table 2, BHJ at the original concentration(1×) showed the DPPH, O_2_^−^, OH and nitrite scavenging activity. The DPPH, O_2_^−^, OH and nitrite radical scavenging rate of BHJ was 34.15 ± 2.18%, 24.35 ± 2.37%, 19.57 ± 1.49% and 38.64 ± 3.42%, respectively.

**Table 2:** The radical scavenging analysis of BHJ

Sample	DPPH scavenging rate(%)	O_2_^­^·scavenging rate(%)	OH radical scavenging rate(%)	Nitrite scavenging rate(%)
BHJ	43.15 ± 2.18%	24.35 ± 2.37%	19.57 ± 1.49%	38.64 ± 3.42%

### Anti-proliferative effect of BHJ

#### BHJ inhibited HepG2 cells viability

**Fig. 1:**
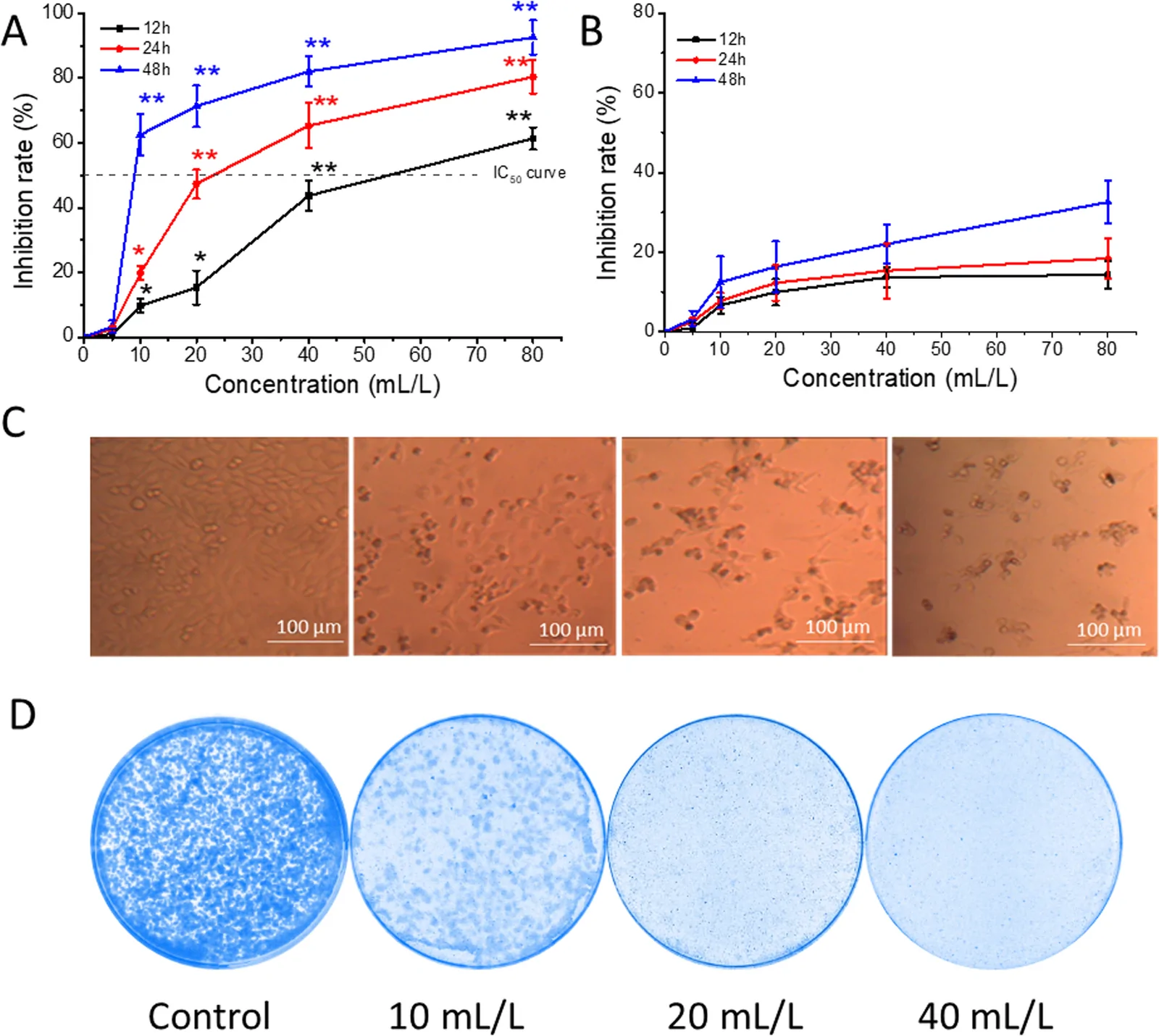
Effects of BHJ on the proliferation of HepG2 cells. **A** Inhibitory effect of BHJ on HepG2 cell growth; **B** Inhibitory effect of BHJ on LO2 cell growth; **C** Morphological analysis of BHJ-treated HepG2 cells; **D** Colony formation assay of BHJ-treated HepG2 cells

BHJ significantly inhibited the proliferation of HepG2 cells in a dose-dependent manner, with half-maximal inhibitory concentration(IC_50_ ) values of 53.21, 21.62 and 8.17 mL/L at 12 h, 24 h and 48 h, respectively (Fig. 1A). Notably, BHJ exhibited minimal toxicity toward normal human liver LO2 cells (Fig. 1B), which supports its potential as a safe and effective candidate for anticancer therapy. Morphological observation revealed that HepG2 cells treated with BHJ displayed typical apoptitic-like changes, including irregular morphology, cellular shrinkage, and rounding (Fig. 1C). Furthermore, BHJ dramatically suppressed the colony-forming ability of HepG2 cells (Fig. 1D). Collectively, these results demonstrate that BHJ possesses potent anti-hepatocellular carcinoma activity.

#### BHJ induced HepG2 cells apoptosis

**Fig. 2:**
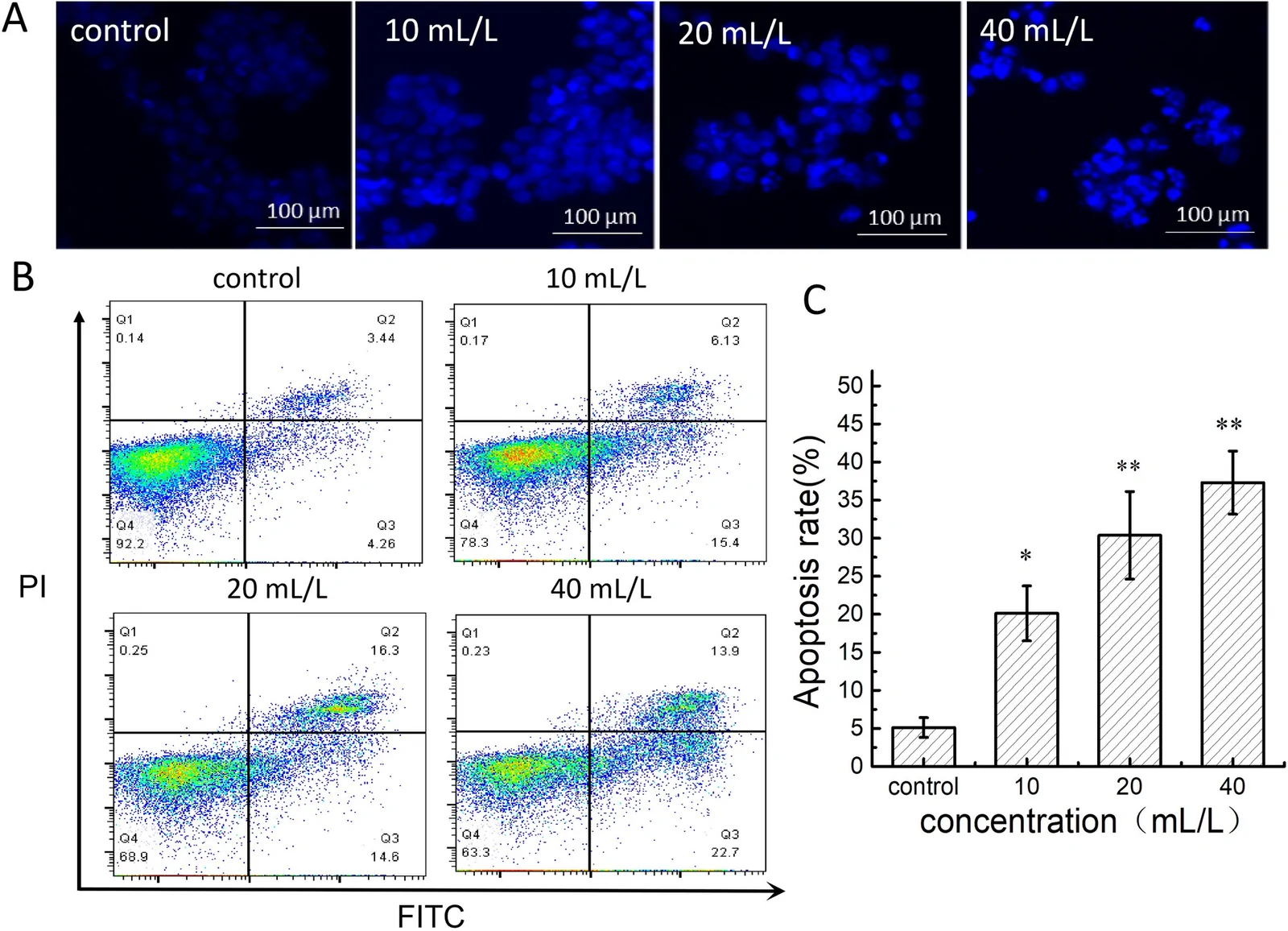
Effects of BHJ on the apoptosis of HepG2 cells. **A** Nuclear morphological changes of HepG2 cells stained with hoechst33258; **B** Representative flow cytometry plots of HepG2 cell apoptosis; **C** Quantitative analysis of apoptotic rates of HepG2 cells. Data are presented as mean ± SD (*n* = 3). *P < 0.05 and **P < 0.01vs. the control group

Nuclear morphological changes were observed under a fluorescent microscope following Hoechst 33,258 staining(Fig. 2A). As shown in Fig. 2A, control HepG2 cells emitted uniform blue fluorescence with consistant intensity, whereas BHJ-treated cells displayed characterstic apoptotic nuclear changes, including chromatin congregated and karyopynosis, which emitted bright blue fluorescence. Further, flow cytometry analysis (Annexin V-FITC/PI double staining) indicated that the apoptotic rates of HepG2 cells in BHJ-treated groups (0, 10, 20, and 40 mL/L) were 3.12 ± 0.3%, 15.12 ± 1.61%, 26.38 ± 1.76% and 31.30 ± 1.15%, respectively. Compared with the control group, the apoptotic rates were significantly increased in the BHJ-treated groups (*P* < 0.05, Fig. 2B, C). These results indicated that BHJ can induce apoptosis of HepG2 cells in a dose-dependent manner.

#### BHJ regulated the expression of apoptotic-related proteins

**Fig. 3:**
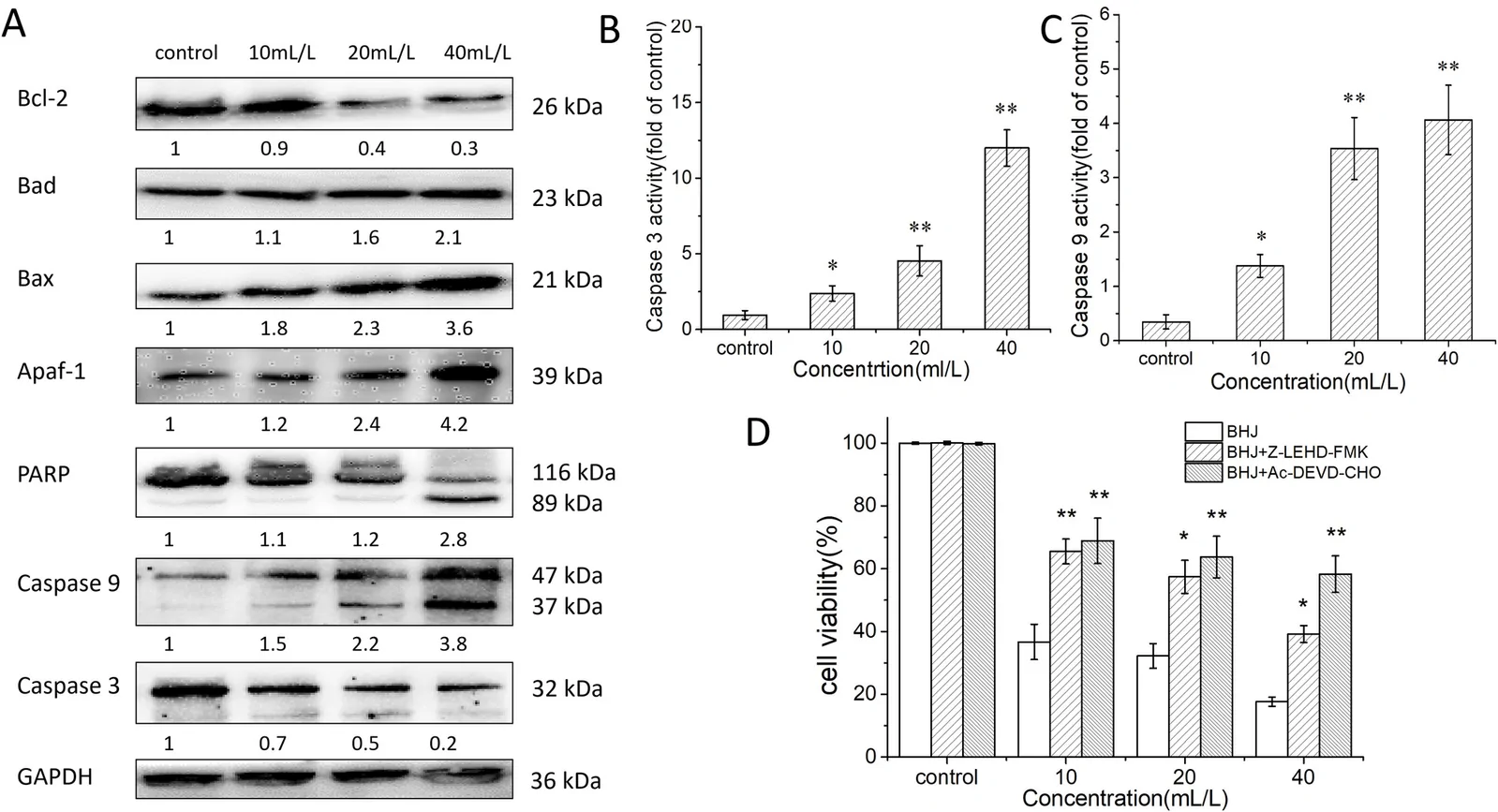
Effects of BHJ on the expression and activity of apototic-related proteins in HepG2 cells. **A** Western blot analysis of apoptotic-associated protein levels in HepG2 cells; **B**, **C** Effect of BHJ on caspase 9, caspase 3 enzyme activities in HepG2 cells; **D** Inhibition of BHJ-induced apoptosis by caspase 9 and caspase 3 inhibitors in HepG2 cells. The values are expressed as means ± SD of triplicates tests. *P < 0.05 comparison between the absence and presence of inhibitor in the treatment group

As shown in Fig. 3, BHJ treatment significantly modulated the expression of key apoptotic-related proteins in HepG2 cells: it up-regulated the expression of pro-apoptotic proteins (Bax and Bad) while down-regulating the anti-apoptotic protein Bcl-2. Additionally, the level of activated caspase 9 was remarkably elevated in BHJ-treated cells. Furthermore, BHJ treatment increased PARP cleavage and Apaf-1 expression (Fig. 3A). Consistent with the protein expression results, the enzymatic activities of both caspase 9 and caspase 3 were markedly enhanced following BHJ treatment (Figs. 3B, C). Notably, pre-treatment with caspase-specific inhibitors-Z-LEHD-FMK(a caspase 9 inhibitor) and Ac-DEVD-CHO(a caspase 3 inhibitor) -significantly restored the viability of BHJ-treated HepG2 cells(Fig. 3D). Collectively, these findings demonstrate that BHJ induces HepG2 cell apoptosis primarily through the mitochondrial- mediated intrinsic apoptotic pathway.

### BHJ Inhibited the migration and Invasion of HepG2 cells

#### Anti-migratory role of BHJ in HepG2 cells

**Fig. 4:**
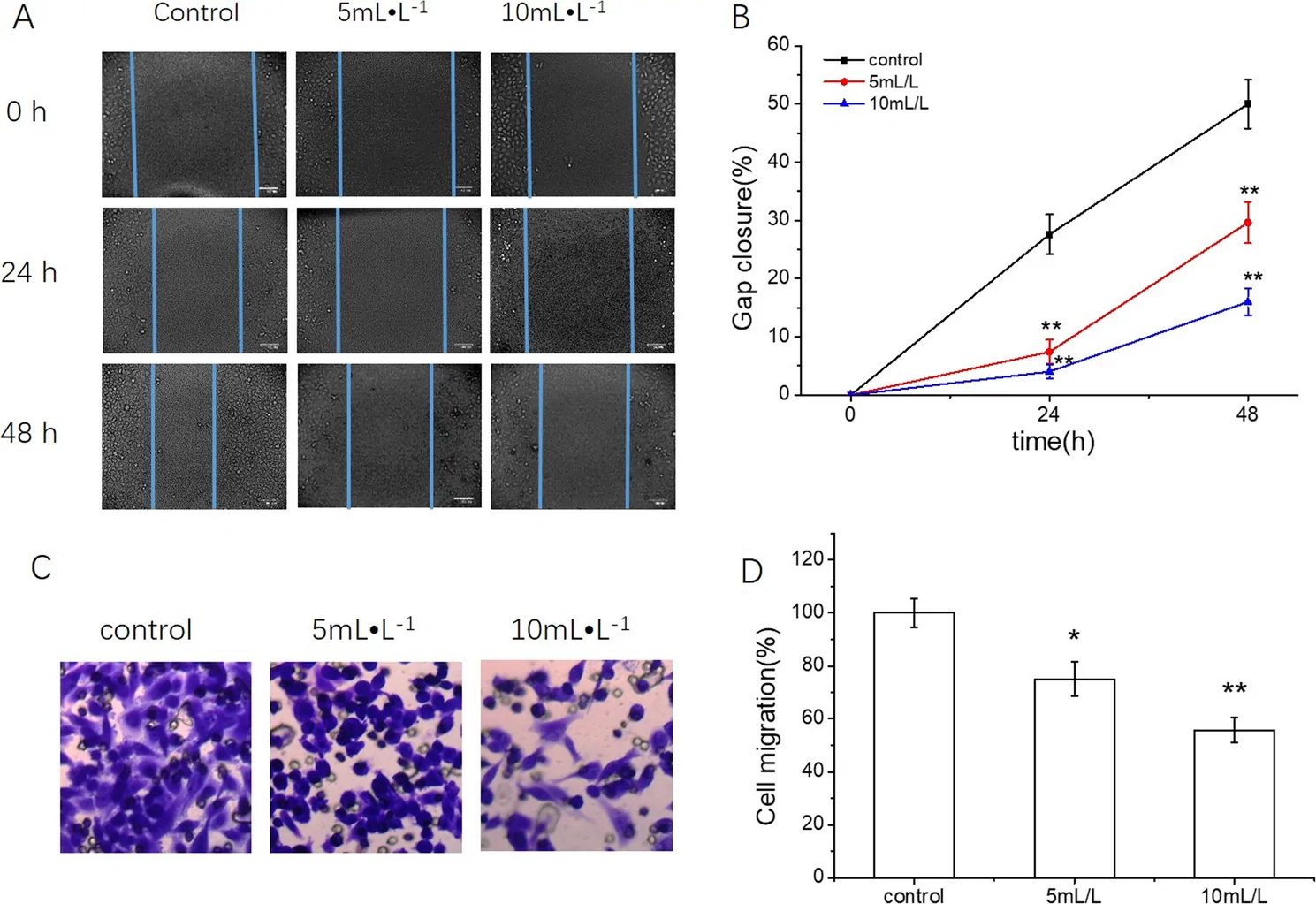
Inhibitory effect of BHJ on cell migration in HepG2 cells. **A** The photomicrographs of migrating HepG2 cells in the wound-healing assay(low magnification, Scale bar = 100 μm). Vertical lines indicate the initial scratch boundary at 0 h and migrating fronts at 24 and 48 h; migration ability is expessed as the percentage of gap closure on the cultured surface. **B** Quantitative analysis of the gap closure rate after BHJ exposure compared with the control group at each time point. **C** Morphology of migrated HepG2 cells stained with crystal violet(inverted microscope, 200× magnification). **D** Histogram shows the percentage of cell migration after incubation with BHJ. Data are expressed as mean ± SD (*n* = 3). *P < 0.05 and **P < 0.01, significant difference from the control group

The wound-healing assay was performed to evaluate the effect of BHJ on HepG2 cell migration, and the results demonstrated significant inhibition following BHJ treatment. As shown in Fig. 4, both 5 and 10 mL/L BHJ significantly suppressed the migration capacity of HepG2 cells compared to the control group after 24 h and 48 h of incubation (Fig. 4A, B). This anti-migratory effect was further validated by transwell migration assays: compared with the control group, HepG2 cell migration was reduced by approximately 24.9% and 44.2% at BHJ concentrations of 5 and 10 mL/L, respectively (Fig. 4C, D). These findings clearly indicate that BHJ effectively inhibits HepG2 cell migration in a dose-dependent manner with statistical significance.

#### Effect of BHJ on HepG2 cell invasion

**Fig. 5:**
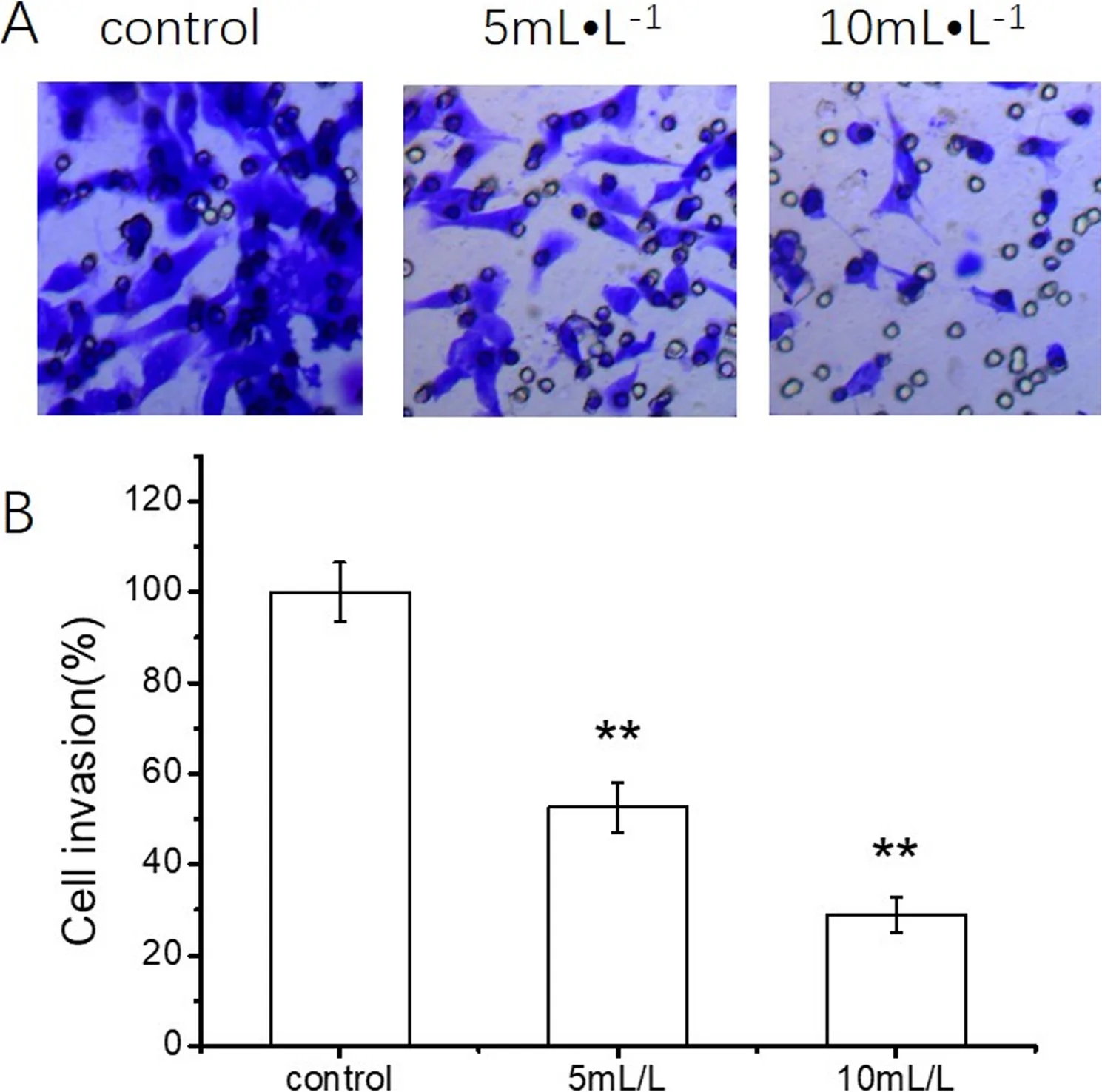
Inhibitory effect of BHJ on cell invasion in HepG2 cells. **A** Morphology of invaded HepG2 cells stained with crystal violet (inverted microscope, 200× magnification). **B** Histogram showing the percentage of invaded cells after incubation with BHJ. Data were expressed as mean ± SD (*n* = 3). *P < 0.05 and **P < 0.01, significant difference from the control group

We further evaluated the anti-invasive potential of BHJ in HepG2 cells using a Matrigel-based transwell assay. As demonstrated in Fig. 5A, representative images of crystal violet-stained invaded cells showed that BHJ treatment significantly inhibited HepG2 cell invasion through the Matrigel-coated membrane compared to untreated controls. Quantitative analysis revealed a dose-dependent inhibitory effect: 5 and 10 mL/L BHJ reduced the invasive capacity of HepG2 cells by 47.5% and 71.2%, respectively (Fig. 5B). These findings provide compelling evidence that BHJ potently suppresses hepatocellular carcinoma cell invasion.

#### Effect of BHJ on MMPs and TIMP expressions

**Fig. 6:**
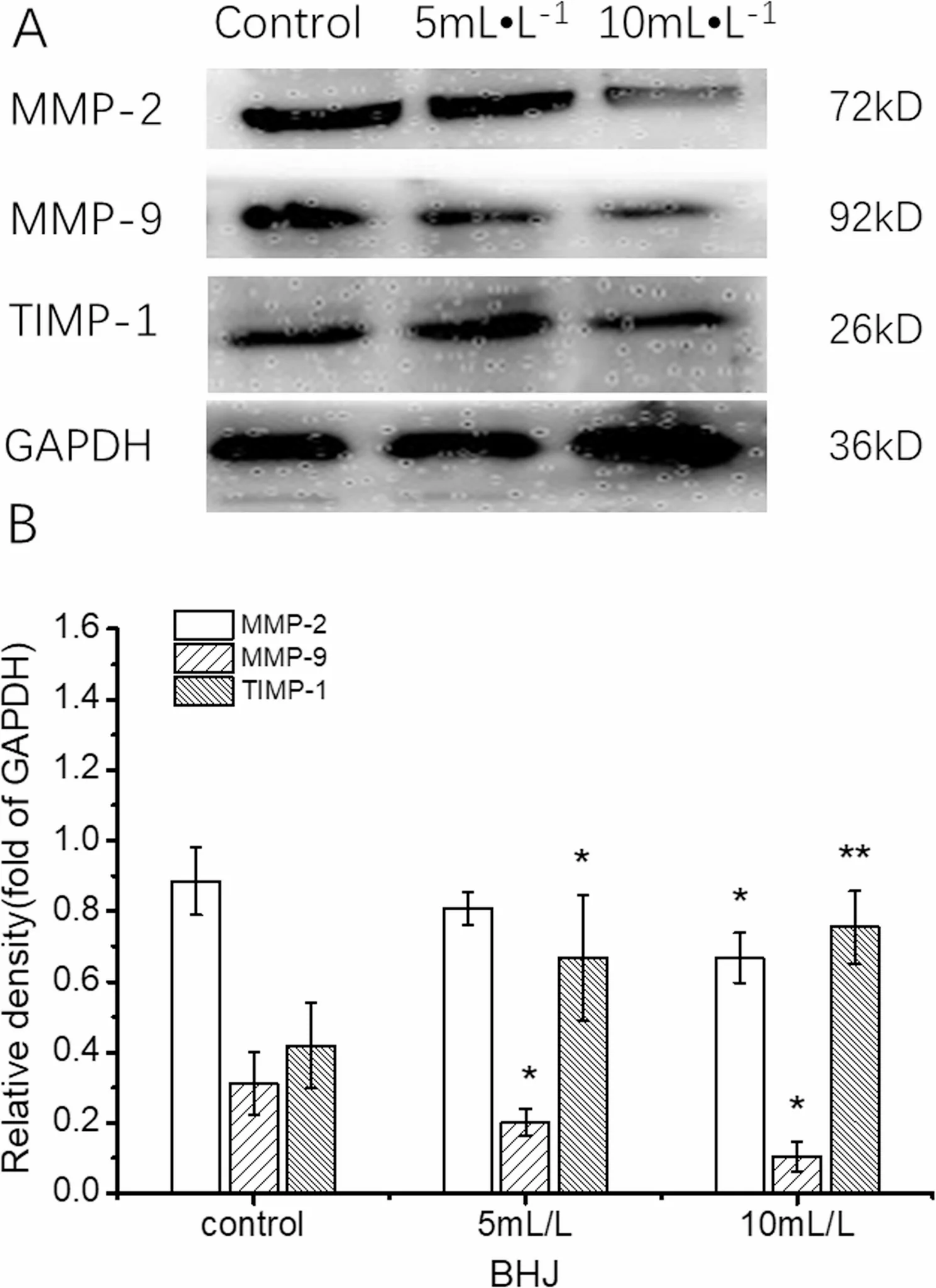
Effect of BHJ on MMPs and TIMP expressions in HepG2 cells. **A** Protein expressions of MMP-9, MMP-2 and TIMP-1 were examined using Western blot analysis. GAPDH was used as the loading control. **B** Relative band intensity was quantified using Image J software and normalized to the control group. Data are expressed as mean ± SD (*n* = 3). *P < 0.05 and**P < 0.01, significant difference from the control group

Western blot analysis was performed to investigate the effect of BHJ on the expression of MMPs and TIMP in HepG2 cells. As shown in Fig. 6, after 48 h of incubation with 10 mL/L BHJ, the expression of metastasis-associated proteins MMP-9 and MMP-2 were significantly downregulated, while the expression of the MMP inhibitor TIMP-1 was notably upregulated(Fig. 6A). Quantitative analysis of relative band intensity confirmed these regulatory effects compared to the control group(Fig. 6B). These findings suggest that BHJ attenuates HepG2 cell migration and invasion by modulating the MMP/TIMP balance, which may be mediated through the inhibition of extracellular matrix degradation.

## Discussion

*Benincasa hispida* Cogniaux, as a food on Chinese tables, also serves as a traditional Chinese herb. Although previous research reported its peel and seeds showed potency against tumors and oxidative stress, no studies have explored the activities of the edible part. In the present study, we investigated the antioxidant and anticancer properties of juice extracted from *Benincasa hispida* Cogniaux (BHJ, excluding the peel and seeds) in HepG2 cells. We found that BHJ demonstrated DPPH, O_2_^−^, OH radical and nitrite scavenging activity and anticancer activity, effectively inducing apoptosis and inhibiting migration and invasion of HepG2 cells without affecting LO2 cells activity.

Many fruits and vegetables harbor a diverse range of bioactive phytochemicals, such as nutritive compounds and also flavonoids, anthocyanins, alkaloids, stilbenes, phenolic acids, and procyanidins [21, 22]. These compounds reportedly contribute to antioxidant plant properties and exhibit anticancer activity [23]. Phenolic and flavonoid contents in vegetables are of particular significance due to their potent antioxidant activity [24]. The unique blend of phytochemicals varies from plant to plant, offering a spectrum of antioxidant activities [25]. Previous studies indicated *Benincasa hispida* peel and seeds also display antioxidant activity due to their total phenolics, flavonoids, and anthocyanins content [26, 27]. Our results showed that BHJ contained 37.14 ± 4.26 mgGAE/L of total phenolics and 12.43 ± 2.36 mgRUT/L juice of flavonoids, and exhibited significant radical scavenging abilities.

Plant-derived extracts have been shown to have profound effects on cancer therapy and prevention [28]. Pomegranate juice promotes apoptosis by induction of mitochondrial dysfunction in HCC cells [29]. Cucurbitacin D from various plant families induced apoptosis and blocked the cell cycle in HepG2 cells [30]. Cucurbitacin E from *Ecballium Elaterium* induced apoptosis in HepG2 cells and halted the cell cycle in the G2/M phase while causing mitochondrial and DNA damage [31]. Bergamot juice reduced the growth rate of human hepatocellular carcinoma HepG2 cells by a mechanism involving the activation of apoptotic machinery via both intrinsic and extrinsic pathways [32]. In accordance with previous reports, our study indicated that BHJ promoted the apoptosis of HepG2 cells via mitochondria-mediated pathway.

Metastasis is significantly pathological characterized by malignant tumors, while the invasion and migration of liver cancer cells lead to prognosis of liver cancer patients, prone to recurrence [33]. The migration and invasion of tumor cells is a complex process controlled by multiple factors, including matrix metalloproteinases (MMPs) [34]. While MMPs are a group of proteolytic enzymes, TIMPs serve as their endogenous inhibitors [35]. Black rice bran‑derived anthocyanins attenuate cholangiocarcinoma cell migration via the alteration of epithelial‑mesenchymal transition [36]. It was reported that kaempferol and doxorubicin synergistically inhibited the proliferation, migration and invasion of liver cancer cells by inhibiting PI3K/mTOR/MMP protein pathway [37]. Cyanidin-3-*O*-glucoside is a potential anticancer reagent as it can inhibit EMT and breast cancer cell migration and invasion [38, 39]. In our study, BHJ could inhibit the migration and invasion of HepG2 cells not only by decreased MMP-9 and MMP-2 expression but also increased TIMP-1 expression.

In summary, our findings highlight the antioxidant and anti-proliferative capabilities of BHJ against liver cancer, majorly by inducing apoptosis and suppressing migration and invasion of HepG2 cells. Notably, BHJ shows no cytotoxicity to normal liver cells, indicating its potential as a safe and effective anti-cancer functional food or auxiliary therapeutic agent-this supports the conclusion that *Benincasa hispida* may serve as a promising source of therapeutic agents for combating oxidative stress and cancer, and also validates its traditional TCM value as a medicine-food homology ingredient for dietary therapy. However, there are still some limitations in this study: the specific active compounds in BHJ that exert antioxidant and anti-cancer effects remain unclear, and the anti-cancer activity of BHJ in other types of cancer cells has not been evaluated. Therefore, further research is required to isolate and identify the active components in BHJ, and to explore its anti-cancer activity and underlying mechanisms in different cancer cell lines and in vivo model, so as to clarify whether the anti-cancer effect of BHJ is cell-line specific or mediated by a universal mechanism—this will lay a foundation for the further development of BHJ as a natural, safe, diet-derived preventive or adjuvant therapeutic option for HCC.

## Supplementary Information

Supplementary Material 1.

## Data Availability

Data is provided within the manuscript or supplementary information files.
