# Supplementary material for: Antioxidant activity and cytotoxic effects of Benincasa hispida juice on HepG2 cells
Source: BMC Complement Med Ther. 2026 Jul 25;26:312. doi: 10.1186/s12906-026-05475-3 (PMC13685248; doi:10.1186/s12906-026-05475-3)
Supplement: Supplementary file 1 [file 12906_2026_5475_MOESM1_ESM.doc]

Figure 3

Croppedblots in main paper	Croppedblots in supplementary information

To ensure the straightness and flatness of the strip, we added a sample on both sides of the target sample, which is a mixture of all remaining target samples(MRTS). The first MRTS: control+10mL/L. The second MRTS: 20 mL/L +40. mL/L

MRTS control 10 20 40 MRTS

29 kD

MRTS control 10 20 40 MRTS

26 kD

MRTS control 10 20 40 MRTS

22 kD

MRTS control 10 20 40 MRTS

21 kD

MRTS control 10 20 40 MRTS

23 kD

MRTS control 10 20 40 MRTS

53 kD

MRTS control 10	20	40	MRTS

130 kD

MRTS control 10	20	40	MRTS

116 kD

89 kD

MRTS control 10	20	40	MRTS

51 kD

37 kD

MRTS control 10	20	40	MRTS

35 kD

MRTS control 10 20	40 MRTS

36 kD

Figure 6

Croppedblots in main paper	Croppedblots in supplementary information

control 5ml/L 10 ml/L control 5 ml/L 10 ml/L

72 kD

control 5ml/L 10 ml/L control 5 ml/L 10 ml/L

92 kD

control 5ml/L 10 ml/L control 5 ml/L 10 ml/L

26 kD

control 5ml/L 10 ml/L control 5 ml/L 10 ml/L

36 kD
